# Combined effect of vitamin C and vitamin D_3_ on intestinal epithelial barrier by regulating Notch signaling pathway

**DOI:** 10.1186/s12986-021-00576-x

**Published:** 2021-05-08

**Authors:** Fubin Qiu, Zehui Zhang, Linxue Yang, Rui Li, Ying Ma

**Affiliations:** Department of Nutrition and Food Hygiene, School of Public Health, Shanxi Medical University, 56 Xinjian South Road, Taiyuan, China

**Keywords:** Intestinal epithelial barrier, Tight junction, Inflammatory bowel disease, Vitamin D_3_, Vitamin C

## Abstract

**Background:**

Tight junction proteins play crucial roles in maintaining the intestinal mucosal barrier. Although previous studies have shown that Notch signaling is closely related to tight junction proteins, the mechanism remains unclear. This study was performed to investigate whether vitamin C combined with vitamin D_3_ affects intestinal mucosal barrier stability via the Notch signaling pathway.

**Methods:**

Intestinal epithelial barrier and notch signaling pathway were studied using guinea pig and SW480 cells. The guinea pigs were randomized into four groups (n = 6 in each group): control group (C, 200 IU/kg d VD_3_ + 100 mg/kg d VC), low VC group (LVC, 200 IU/kg d VD_3_ + 10 mg/kg d VC), medium VC group (MVC, 200 IU/kg d VD_3_ + 100 mg/kg d VC), and high VC group (HVC, 200 IU/kg d VD_3_ + 200 mg/kg d VC). Except for the control group, the other three groups were freely drinked with 2% dextran sodium sulfate solution for 4 days. And the control group was free to drink distilled water. The following cell groups were used: control group (SW480 cells without intervention); LPS group (100 ng/mL LPS); VD_3_ group (0.1 μmol/L VD_3_); VC + VD_3_ group (0.1, 1, 5, 10 μmol/mL VC + 0.1 μmol/L VD_3_).

**Results:**

Electron microscopy analysis revealed that both low and high doses of vitamin C combined with vitamin D_3_ maintained dextran sodium sulfate-induced ulcerative colitis in the guinea pig intestinal epithelium tight junction. Compared with the control group, the expression level of ZO-1 mRNA in the colon tissue of the high-dose vitamin C group was significantly increased. In SW480 cell experiments, compared with the control group, cell migration and repair following treatment with different concentrations of vitamin C combined with vitamin D_3_ were significantly improved and the protein expression of Notch-1 was increased, whereas the protein expression of claudin-2 was significantly decreased. Thus, our results demonstrate that an appropriate amount of vitamin C combined with vitamin D_3_ can regulate the expression of claudin-2 by regulating Notch-1, relieve destruction of the intestinal mucosal barrier, and promote the repair of damage to the cell mucosal barrier.

**Conclusions:**

We found that vitamin C combined with vitamin D_3_ protected against dextran sodium sulfate-induced ulcerative colitis in the guinea pig intestinal mucosa.

## Introduction

Ulcerative colitis (UC) is an inflammatory bowel disease (IBD) that causes irritation, inflammation, and ulcers in the lining of the large intestine or colon. The pathogenesis of UC disease is not fully understood but is known to be related to genetic susceptibility, immunity, and other factors such as abnormal expression of cytokines, an imbalance in the intestinal flora, diet, and infection [1–3]. Because the disease is chronic and prone to recurrent episodes, patients with UC have an increased risk of colon cancer [4, 5]. As an important interface between the body and external environment, the intestinal mucosal barrier plays a key role in maintaining intestinal homeostasis. Many studies have shown that intestinal mucosal damage is the initiating factor of IBD. The intestinal mucosa constitutes an important barrier, and thousands of microorganisms and environmental antigens are in close contact with the host immune system [6]; damage to the intestinal mucosa results in increased mucosal barrier permeability, and a large number of bacteria in the intestine are absorbed into the blood, in turn inducing an intestinal immune response [7, 8]. Therefore, protecting and repairing the intestinal mucosal barrier is key to preventing the development of IBD.

The intestinal mucosal barrier is composed of epithelial cells, tight junction proteins, and intestinal secretions [9], with tight junction proteins acting as the most important connection between intestinal mucosal cells and exerting important roles in maintaining intestinal barrier integrity. The tight junction complex mainly includes claudin, zona occludens (ZO) family, and other proteins. The expression and distribution of claudin are closely related to the permeability of endothelial cells. ZO protein is a backbone protein that helps link the tight junctions to each cell cytoskeleton [10], and its normal expression is an important condition for stabilizing the cell structure. In contrast, abnormal expression of ZO may destroy the structure of epithelial and endothelial cells, resulting in impaired function. Studies have shown that increased expression of the Notch1 receptor and downstream gene Hes1 can promote repair of the injured epithelium, suggesting that the Notch1 signaling pathway is involved in regulating the proliferation and differentiation of epithelial cells [11].

Studies have shown that the active form of vitamin D_3_ (1,25 (OH)_2_D_3_) can regulate the expression of family proteins such as claudin and ZO in the tight junction complex, which is important for maintaining and repairing the mucosal barrier [12, 13]. Additionally, nutritional supplementation with vitamin C has been demonstrated to have beneficial effects on IBD [14].

In this study, we investigated whether vitamin C combined with vitamin D_3_ mediates the Notch signaling pathway in protecting the intestinal mucosal barrier. We evaluated vitamin C combined with vitamin D_3_ in in vitro and in vivo intervention experiments in a UC model to explore whether combining these vitamins has greater protective effects on the intestinal mucosal barrier and to explore the role of the Notch signaling pathway in the intestinal mucosal barrier.

## Methods and materials

### Design of animal experiment

#### Animal model and groups

All animal procedures were approved by the Institutional Animal Care and Use Committee of Shanxi Medical University and conformed with Guide for the Care and Use of Laboratory Animals 8th Edition 2011.

Twenty-four male Dunkin–Hartley guinea pigs (360 ± 20 g; Longan Experimental Animal Breeding Center, Beijing, China) were housed in metabolic cages at an ambient temperature of 22 ± 1 °C with a relative humidity of 55–60% and 12-h/12-h yellow light/dark cycle. Guinea pigs initially received distilled water and regular diets (Beijing Keao Xieli Feed Co, Ltd., Beijing, China), a standard chow for laboratory guinea pigs (GB 14924.3-2010). Adaptive feeding was carried out for 1 week, after which the guinea pigs were randomized into 4 groups (n = 6 in each group): control group (C, 200 IU/kg d VD_3_(VD_3_, Vitamin D_3_) + 100 mg/kg d VC(VC, Vitamin C)), low VC group (LVC, 200 IU/kg d VD_3_ + 10 mg/kg d VC), medium VC group (MVC, 200 IU/kg d VD_3_ + 100 mg/kg d VC), and high VC group (HVC, 200 IU/kg d VD_3_ + 200 mg/kg d VC). Guinea pigs in each group were fed a purified diet without vitamin D_3_ or vitamin C (Research Diets, New Brunswick NJ, USA) (Table 1), and the corresponding doses of VC and VD_3_ were administered by gavage.

**Table 1:** Feed formulations that do not contain VC and VD_3_

	D17022201	
	g%	kcal%
*Product #*		
Protein	17.6	20
Carbohydrate	60.2	69
Fat	3.9	10
Total		99
kcal/g	3.50	
*Ingredient*		
Soy protein	80	320
Corn starch	315	1260
Maltodextrin 10	35	140
Sucrose	350	1400
Cellulose, BW200	100	0
Guar gum	25	0
Lard	20	180
Soybean oil	25	225
Mineral mix S20001	75	0
Vitamin mix V23903 (without vitamin D_3_ and vitamin C)	10	40
Choline bitartrate	2	0

For pretreatment of vitamin C solution, we took 8 g of VC, added 40 mL of distilled water to dissolve to prepare a final concentration of 200 mg/mL VC solution, and then used distilled water for gradient dilution to the final concentration of 100, 10 mg/mL VC solution, ready to use. For pretreatment of vitamin D, we took 2 mg of VD_3_ powder, added 2 mL of absolute ethanol to dissolve it, then added 398 mL of normal saline to dilute to a final concentration of 200 IU/mL VD_3_ solution, store at 4 °C in the dark, prepare once a week.

After 5 weeks, the control group continued to drink distilled water, and the other 3 groups were treated freely with 2% dextran sodium sulfate (DSS) solution for 4 days, and the DSS was dissolved in distilled water. All guinea pigs were deeply anesthetized by intraperitoneal injection with 3% pentobarbital solution, and then sacrificed by exsanguination.

#### Reagents

DSS (Mr: 36,000–50,000) was purchased from MP Biomedicals (Santa Ana, CA, USA). Vitamin C and Vitamin D_3_ were purchased from Beijing Solarbio Science & Technology Co., Ltd. (Beijing, China).

#### Hematoxylin–Eosin staining of the colon tissue

The colon tissue was fixed with neutral-buffered formalin for 24 h, and then paraffin sectioning and hematoxylin–eosin staining were performed. Histological sections was observed by microscope and severity of inflammation was evaluated by histosome pathology scoring criteria. Table 2 for specific scoring criteria.

**Table 2:** Gross morphological damage score criteria for colonic tissue by naked eye

Gross morphology	Score
No hyperemia, edema, and ulcers	0
Only mucous erythema	1
Mild mucosal edema, slight bleeding	2
Moderate edema, hemorrhagic ulcer, erosion	3
Severe ulcers, erosions, edema	4

#### Electron microscopic study of guinea pig colon

For electron microscopy, 1-cm sections of the gut segments were obtained from the colon 3 cm proximal to the ileo-cecal valve. The gut samples were rinsed with ice-cold saline (0.9% NaCl) and then cut into square-fragments (1 cm^2^) which were placed in cold 2% glutaraldehyde stationary solution for fixation. After fixing the samples, the colon structure was observed and images were obtained under an electron microscope (JEOL 1011, OLYMPUS, Japan).

#### RT-qPCR for Notch-1, Hes-1, Claudin-2, and ZO-1

Total RNA was extracted from colon tissue samples of guinea pigs using an Eastep® Super Total RNA Extraction Kit (Promega, Madison, WI, USA) according to the manufacturer’s protocol. An equal amount (0.5 μg) of mRNAs was converted to cDNA using a reverse transcription kit (Promega). RT-qPCR was performed using 2 μL cDNA with a Reverse Transcription Kit (Promega). GoTaq qPCR Master Mix (Promega) was used for RT-qPCR analysis on the CFX96TM Real-Time System (Bio-Rad, Hercules, CA, USA).

Guinea pig mRNA sequences were used to design primer pairs for RT-qPCR with Oligo Primer Analysis Software. The primer sequences are shown in Table 3 and the primers were obtained from Thermo Fisher Scientific (Waltham, MA, USA). GAPDH/β-actin was used as the endogenous loading control. Gene expression was determined using the delta–delta Ct method: 2^−ΔΔCT^ (ΔΔCT = [Ct (target gene) − Ct (GAPDH/β-actin)]_experiment_ − [Ct (target gene) − Ct (GAPDH/β-actin)]_control_).

**Table 3:** Polymerase chain reaction primers' gene sequences for guinea pigs

Species	Target gene	Primer sequence	Length (bp)
Guinea pigs	β-Actin	Forward:CCACCATGTACCCAGGCATT	177
		Reverse:ACTCCTGCTTGCTGATCCAC	
	GAPDH	Forward:ACGGATTTGGCCGTATTGGA	142
		Reverse:GGAACTTGCCGTGGGTAGAA	
	Notch1	Forward:AAAGACCTCAAGGCACGGAG	222
		Reverse:CAGATGGTTGATGCCCAGGT	
	Hes-1	Forward:AAAGTATTCGGCGGCTTCCA	154
		Reverse:TGGAAGGAGATACGGCGTTG	
	Claudin-2	Forward:TCATGGGATTTTGCGGGACT	158
		Reverse:TGGCACGATTTCCTTGGGAT	
	ZO-1	Forward:CCAGTCCCTTACCTTTCGCC	196
		Reverse:GTGACGGCTCTTGGTCTCTT	

### Cell culture

#### Cells culture, grouping, and collection

Human colon cancer SW480 cells were obtained from Shanxi Provincial Cancer Hospital. The SW480 cells were cultured in Dulbecco’s Modified Eagle Medium containing 10% (v/v) heat-inactivated fetal bovine serum (EVERY GREEN, Zhejiang Tianhang Biotechnology Co., Ltd., Zhejiang, China), penicillin (100  U/mL), and streptomycin (100 μg/mL) (BOSTER, Wuhan, China) in a humidified atmosphere of 5% CO_2_ and 95% air at 37 °C. SW480 cells were seeded at 10^4^ cells per well. After 24 h, except for the control group, lipopolysaccharide (LPS; 100 ng/mL) was added to the cells followed by incubation for 24 h to establish an in vitro model of inflammatory bowel disease. Cell proliferation was quantified using the Cell Counting Kit-8 (BOSTER).

The following cell groups were used: control group (without intervention); LPS group (100 ng/mL LPS); VD_3_ group (0.1 μmol/L VD_3_); VC + VD_3_ group (0.1, 1, 5, 10 μmol/ml VC + 0.1 μmol/L VD_3_).

#### Cell scratch test

SW480 cells in the logarithmic growth phase were divided into a normal control group (C, No VC, VD added), VD_3_ group (0.1 μmol/L VD_3_), and VC + VD_3_ group (0.1, 1, 5, or 10 μmol/mL VC + 0.1 μmol/L VD_3_) with 2 duplicate wells per group. A 6-well culture plate was inoculated with the cells at a density of 1–4 × 10^5^ cells per well and cultured for 24 h. Without changing the medium, a 200-μL pipette tip was used to scribe a line across the entire well. The medium was discarded, and the cell surface was washed three times with sterile phosphate-buffered saline. Medium containing no fetal bovine serum was added to each group. Except for the normal control group, the other groups were treated with the corresponding doses of VC and VD_3_ (VD_3_ group (0.1 μmol/L VD_3_), and VC + VD_3_ group (0.1, 1, 5, or 10 μmol/mL VC + 0.1 μmol/L VD_3_). The trace widths of the different treatment groups were measured.

The cell scratch test is a method for determining the migration and repair ability of cells. Therefore, to observe the effect of different concentrations of vitamin C combined with vitamin D_3_ on the migration of SW480 cells, we performed a cell scratch test.

#### Western blotting analysis of Claudin-2, Hes-1, Notch-1, and ZO-1

SW480 cells were lysed in lysis buffer containing Proteinase Inhibitor Cocktail (KeyGEN BioTECH, Nanjing, China) and Halt Phosphatase Inhibitor Cocktail (KeyGEN BioTECH). Protein concentrations were quantified with a BCA protein kit (KeyGEN BioTECH). Samples (30 μg protein per lane) were loaded into 8–10% SDS–PAGE gels. After electrophoresis, the proteins were transferred from the gel to polyvinylidene fluoride membranes. The membranes were blocked with 5% skim milk and incubated at 4 °C overnight with primary antibodies [β-actin polyclonal antibody (Bioworld, Irving, TX, USA; 1:5000), Claudin-2 antibody (Affinity, Cincinnati, OH, USA;1:500), Hes-1 polyclonal antibody (Abclonal, Wuhan, China; 1:500), Notch-1 polyclonal antibody (Abclonal, 1:500), and ZO-1 polyclonal antibody (Abclonal, 1:200)]. Immunoreactivity was detected by incubation with horseradish peroxidase-conjugated secondary antibodies (BOSTER, 1:2000) followed by chemiluminescent substrate development (Affinity). The results were detected by using a ChemiDoc XRS + with Image Lab Software (Bio-Rad). All samples were evaluated in parallel with three replicates.

#### RT-qPCR for Notch-1, Hes-1, Claudin-2, and ZO-1

Total RNA was extracted from SW480 cell samples using the Eastep® Super Total RNA Extraction Kit (Promega) according to the manufacturer’s protocol; other steps were performed as described above. The gene primer sequences required for SW480 cells are shown in Table 4.

**Table 4:** Polymerase chain reaction primers' gene sequences for homosapiens

Species	Target gene	Primer sequence	Length (bp)
Homosapiens	β-Actin	Forward:GAGAAAATCTGGCACCACACC	177
		Reverse:GGATAGCACAGCCTGGATAGCAA	
	GAPDH	Forward:CCTGCACCACCAACTGCTTA	176
		Reverse:GGCAGGGATGATGTTCTGGA	
	Notch1	Forward:TGAATGGCGGGAAGTGTGAAG	219
		Reverse:CATTGTCCAGGGGTGTCAGG	
	Hes-1	Forward:AAAAATTCCTCGTCCCCGGT	194
		Reverse:TGCCGCGAGCTATCTTTCTT	
	Claudin-2	Forward:TATGTCGGTGCCAGCATTGT	257
		Reverse:GCTACCGCCACTCTGTCTTT	
	ZO-1	Forward:AGCCATTCCCGAAGGAGTTG	199
		Reverse:AGGATCACCGTCAGGAGTCA	

### Statistical analysis

Values are presented as the means ± standard deviation ($\overline{\mathrm{x} }$ ± standard deviation). Normally distributed data were compared by one-way analysis of variance, least significant difference*-t* (homogeneous variance), or Dunnett’s T3 (heterogeneous variance) test. The rank sum test was used for non-normally distributed data and Spearman correlation test was used for correlation analysis. The results were considered significant at *P* < 0.05. Statistical tests were performed with SPSS 22.0 software (SPSS, Inc., Chicago, IL, USA). GraphPad Prism version 8.0 software (GraphPad, Inc., La Jolla, CA, USA) was used to generate histograms.

## Results

### VC + VD_3_ promotes recovery of DSS-induced colitis in guinea pigs

**Fig. 1:**
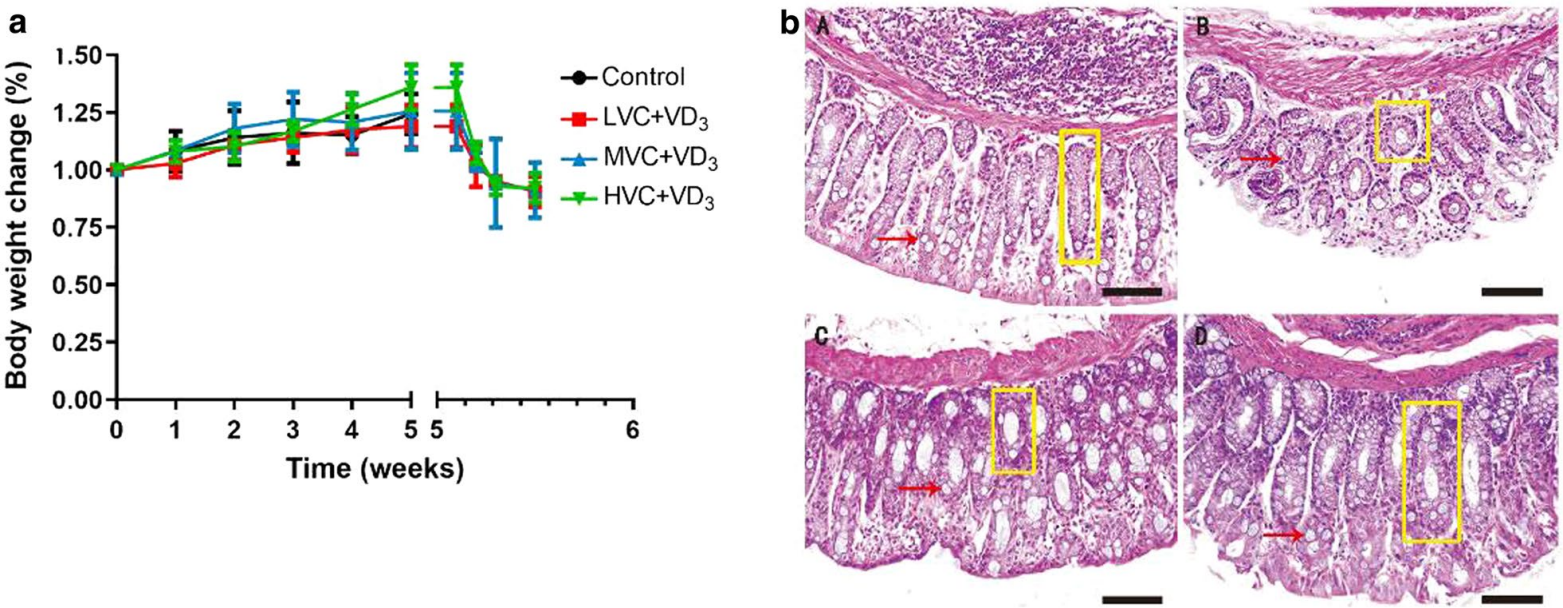
VC + VD_3_ promotes recovery of DSS-induced colitis in guinea pigs. Except for the control group, DSS (2% w/v) was administered for 5 days to guinea pigs in the LVC + VD_3_ group, MVC + VD_3_ group, and HVC + VD_3_ group. **a** Body weight change; changes in body weight in each group of guinea pigs were compared to those in the same group on the 1st day. **b** Histologic images of guinea pig colon (hematoxylin and eosin, magnification × 200, scale bar represents 100 μm). **a** Control group; **b** LVC + VD_3_ group (Low-dose VC; 200 IU/(kg d) VD_3_ + 10 mg/(kg d)); **c** MVC + VD_3_ group (Middle-dose VC; 200 IU/(kg d) VD_3_ + 100 mg/(kg d)); **d** HVC + VD_3_ group (High-dose VC; 200 IU/(kg d) VD_3_ + 200 mg/(kg d)). Red arrows indicate goblet cells; yellow boxes indicates the epithelial crypt

After adaptive feeding of the guinea pigs for 5 weeks, they were treated with 2% DSS for 4 days to induce acute colitis. At the beginning of the experiment, the guinea pigs in each group were in good mental health. There was no significant difference in body weight among groups. In the low, medium, and high dose VC groups, the weight of the guinea pigs began decreasing on the first day after drinking 2% DSS solution. Starting on the second day, the guinea pigs gradually showed signs of apathy, decreased mobility, loose stools, mucus, bloody stools, etc., indicating that preparation of the UC model was successful. After modeling, the body weight of each group of guinea pigs showed a downward trend, but the differences between groups were not significant (*P* > 0.05) (Fig. 1a). Compared with the control group, the colonic gross morphology score and histopathological score of the other groups were significantly increased (*P* < 0.05). Compared with the medium dose VC group, the colon length of the high dose VC group was significantly increased (*P* < 0.05) (Table 5). Compared with the control group, the hematoxylin and eosin staining showed epithelial crypt damage and severe mucosal inflammation in model low-dose VC, middle-dose VC, high-dose groups. In the low-dose VC group, a small number of epithelial cells were exfoliated in the colon, the mucosal surface was finger-like, and the crypt opening was widened; these effects were less severe than those in the medium-dose VC group. The middle-dose VC group showed severe epithelial cell shedding in the guinea pig colon, and the mucosal surface the villous were altered, the crypt had shrunk, the spacing increased, and the connective tissue in the submucosa was loose. The mucus in the colonic goblet cells of the guinea pigs in the high-dose VC group was decreased and the crypts were branched and twisted, but epithelial cell shedding was improved compared to the medium-dose VC group (Fig. 1b).

**Table 5:** Effect of VC + VD_3_ on DSS-induced colonic morphology in guinea pigs ($\overline{x }$ ± s, n = 6)

Group	Length of colon (cm)	Colonic gross morphological score	Colonic histopathological score
Control	60.80 ± 12.73	0.00 ± 0.00	0.00 ± 0.00
LVC + VD_3_ + DSS	60.90 ± 10.76	2.80 ± 0.83^a^	3.50 ± 0.55^a^
MVC + VD_3_ + DSS	52.00 ± 7.77	2.00 ± 0.63^a^	4.00 ± 0.71^a^
HVC + VD_3_ + DSS	64.70 ± 5.25^b^	2.50 ± 0.57^a^	3.50 ± 0.84^a^
*P* value	0.148	0.000	0.000

^a^*P* indicates compared with the control group, *P* < 0.05; the ^b^*P* expression compared with the MVC group, *P* < 0.05

#### VC + VD_3_ attenuate DSS-induced epithelial permeability through regulates colonic tight junction

The DSS model not only affects the integrity of the mucosal barrier, but also regulates colonic tight-junctions. Damage to intestinal permeability may be due to dysregulation of the mucus or epithelial junction complex, which enhances the susceptibility to colitis.

**Fig. 2:**
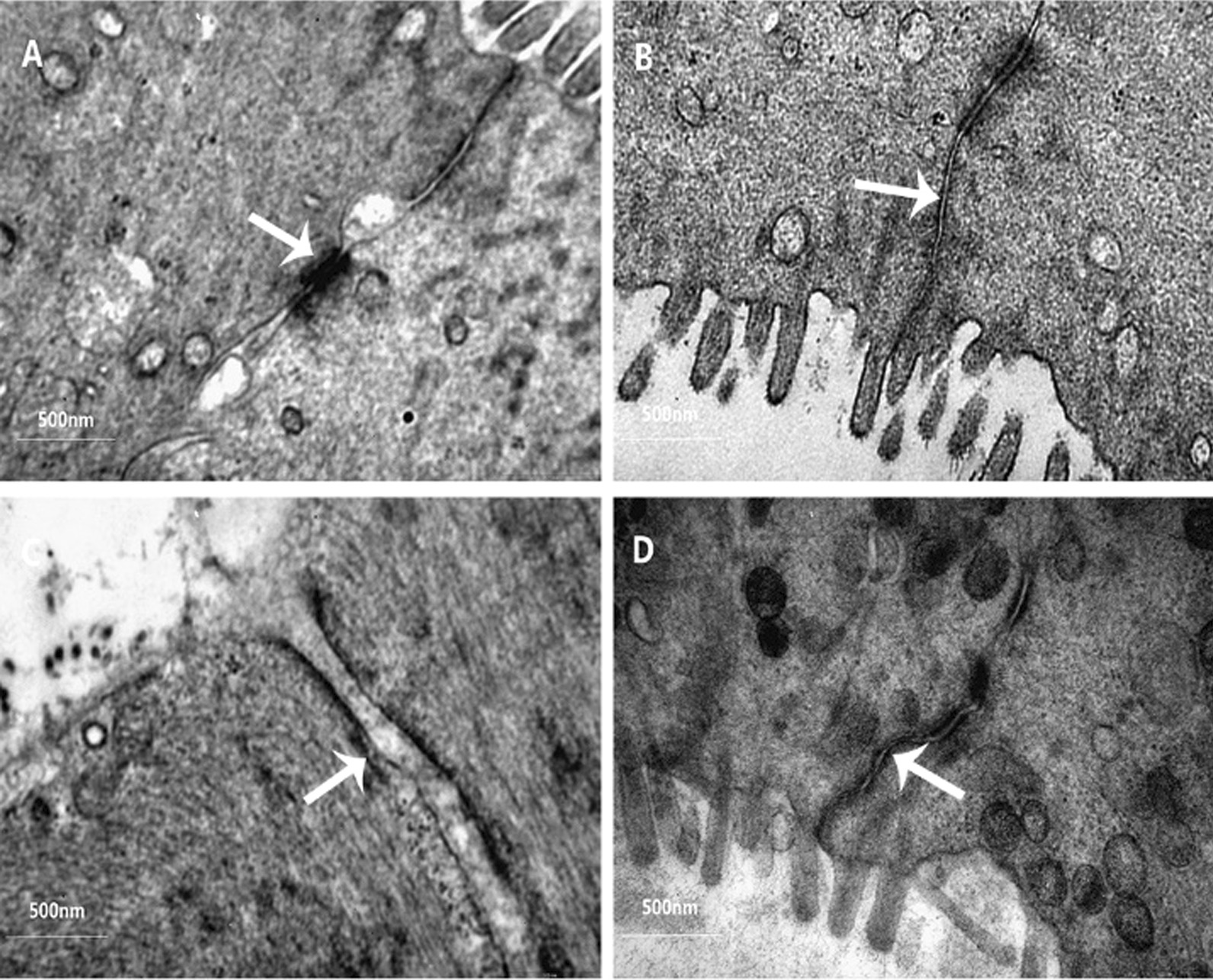
Tight-junction of colonic epithelial cells of guinea pigs under electron microscope (60,000 ×). **a** Control group, **b** LVC + VD_3_ group, (low-dose VC; 200 IU/(kg d) VD_3_ + 10 mg/(kg d)); **c** MVC + VD_3_ group (middle-dose VC; 200 IU/(kg d) VD_3_ + 100 mg/(kg d)); **d** HVC + VD_3_ group (high-dose VC; 200 IU/(kg d) VD_3_ + 200 mg/(kg d)). The white arrows indicate tight junctions

Previous reports suggested that both vitamin D_3_ and vitamin C play a role in maintaining the integrity of the intestinal mucosal barrier and repairing the mucosal barrier [12, 13, 15]. According to our results, there was no significant change in the ultrastructure of colonic epithelial cells in the control group (Fig. 2a). The low, medium, and high dose groups of guinea pig colonic epithelial cells in the VC group showed focal reduction, shortening, and sparseness to different degrees. The medium dose VC group showed the most severe damaged (Fig. 2c). Compared with the control group, the tight junction structure between colonic epithelial cells in the medium-dose VC group was severely damaged and the cell gap became larger (Fig. 2c), whereas the tight junction gap between the colonic epithelial cells in the low-dose and high-dose VC group was slightly widened but the tight joint structure remained essentially intact (Fig. 2b, d).

#### VC + VD_3_ regulate Notch signaling in the colon of DSS-treated guinea pigs

Notch signaling pathway plays a key role in the fate of intestinal epithelial cells [16], which regulate numerous genes including Hes-1 to promote repair of the damaged epithelium [11]. Therefore, the Notch signaling pathway may play an important regulatory role in the maintenance of intestinal mucosal homeostasis by regulating the proliferation and differentiation of intestinal epithelial cells.

We found that the expression levels of Notch1 and Hes1 mRNA in the LVC group were significantly higher than those of the control group (*P* < 0.05) but the expression levels of the middle-dose and high-dose groups were lower than those in the low dose group (*P* < 0.05). The expression distribution of tight junction complexes is closely related to endothelial cell permeability. The results of this experiment show that compared with the control group, the expression level of ZO-1 mRNA in the high-dose group was significantly increased (*P* < 0.05) (Table 6). However, the mRNA expression level of claudin-2 was not significantly different among groups.

**Table 6:** Relative mRNA expression of Notch1 Hes1, claudin2, and ZO-1 in colon tissue ($\overline{x }$ ± s, n = 6)

Group	Notch-1	Hes1	Claudin-2	ZO-1
Control	1.46 ± 1.27	1.17 ± 0.83	2.74 ± 2.26	1.11 ± 0.60
LVC + VD_3_ + DSS	318.88 ± 202.43^a^	9.68 ± 6.94^a^	1.26 ± 1.69	5.38 ± 3.27
MVC + VD_3_ + DSS	3.29 ± 3.11	0.53 ± 0.22^b^	1.49 ± 0.40	6.42 ± 1.21
HVC + VD_3_ + DSS	1.12 ± 1.53^b^	0.34 ± 0.11^b^	1.25 ± 1.24	8.71 ± 4.90^a^
*P* value	0.011	0.030	0.619	0.077

^a^*P* indicates compared with the control group, *P* < 0.05; ^b^ *P* expression is compared with the LVC + VD_3_ group, *P* < 0.05.

### Repair effect of VD_3_ combined VC on tight connection damage between SW480 cells induced by LPS by Notch signal pathway

#### Cell scratch test

**Fig. 3:**
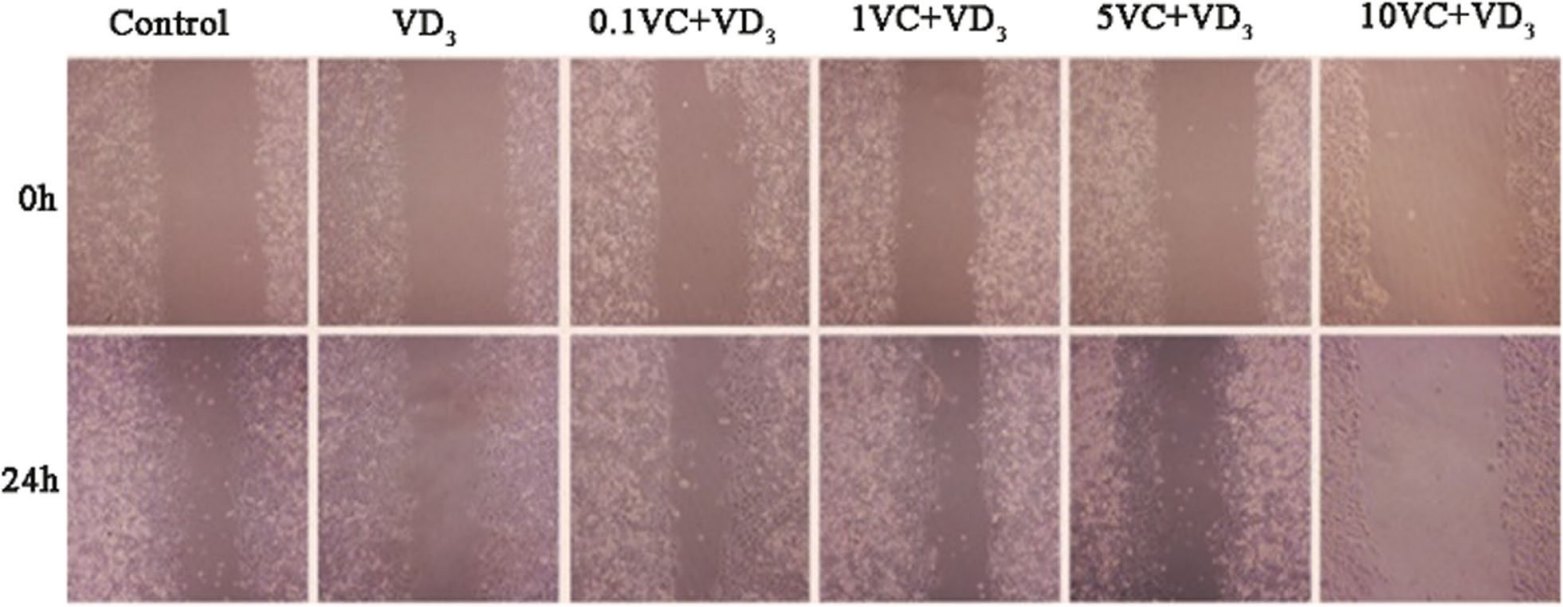
Cell scratch test for SW480 cells (200 ×). SW480 cells in logarithmic growth phase were divided into normal control group (C), VD_3_ group (0.1 μmol/L VD_3_), VC + VD_3_ group (0.1, 1, 5, 10 μmol/mL VC + 0.1 μmol/L VD_3_)

Compared with cell scratches at 0 h, cell migration was observed in all groups at 24 h after cell scratching. Among them, the 0.1VC + VD_3_ group showed the most significant cell migration compared to the control group, whereas the 10VC + VD_3_ group showed the least cell migration. Compared with the VD_3_ group, the cell migration and repair ability of the 0.1VC + VD_3_, 1VC + VD_3_, and 5VC + VD_3_ groups were significantly improved (Fig. 3).

#### VC + VD_3_ attenuate LPS-induced tight junction structure damage to SW480 cells

**Fig. 4:**
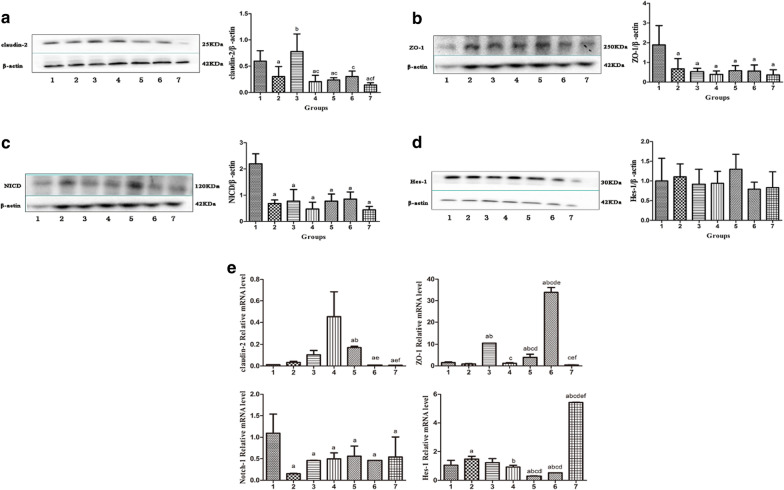
Expression of tight junction-associated proteins and genes and Notch signaling pathway-related proteins and genes in SW480 cells. 1: control group; 2: LPS group; 3: VD_3_ group; 4: 0.1VC + VD_3_ group; 5: 1VC + VD_3_ group; 6: 5VC + VD_3_ group; 7: 10VC + VD_3_ group. **a** Western blotting results of claudin-2 in SW480 cells, **b** western blotting results of ZO-1 in SW480 cells, **c** western blotting results of Notch intracellular domain (NICD) in SW480 cells, **d** western blotting results of Hes-1 in SW480 cells, **e** RT-PCR of tight junction protein-related genes and Notch signaling pathway-related genes. In the figure, **a**–**f** indicate significant differences compared with the control group, LPS group, VD_3_ group, 0.1VC + VD_3_ group, 1VC + VD_3_ group, and 5VC + VD_3_ group, respectively

Tight junctions are essential for the mucosal barrier; therefore, an LPS in vitro model was used to induce cell membrane barrier destruction, and the effects of different doses of vitamin C and vitamin D_3_ on cell tight junctions were observed. As marker of the tight junction structure, the distribution and expression of claudin-2 and ZO-1 were detected by western blotting. Compared with the control group, the expression level of claudin-2 protein was significantly decreased in the LPS, 0.1VC + VD_3_, 1VC + VD_3_, and 10VC + VD_3_ groups (*P* < 0.05), and the expression level of claudin-2 protein in the VD_3_ group was significantly higher than that in the LPS group (*P* < 0.05). The expression level of claudin-2 protein in the VC + VD_3_ group was significantly lower than that in the VD_3_ group (*P* < 0.05). Western blot analysis revealed decreased expression of ZO-1 in LPS group, VD_3_ group, 0.1VC + VD_3_ group, 1VC + VD_3_ group, 5VC + VD_3_ group, and 10VC + VD_3_ group vs the control group (*P* < 0.05) (Fig. 4). Thus, VC + VD_3_ in the intestinal epithelium may enhance epithelial tight junctions in SW480 cells.

Compared with the control group, the mRNA expression levels of Notch-1 (NICD) in the other groups were significantly reduced (Fig. 4e). Compared with the control group, the Hes-1 mRNA expression level of the LPS group and the 10C + D group was significantly increased (Fig. 4e), and the Hes-1 mRNA expression level of the 10C + D group was significantly higher than the LPS group. Compared with the LPS group, Hes-1 mRNA expression levels in the 0.1C + D, 1C + D, and 5C + D groups were significantly lower (Fig. 4e).

## Discussion

Both guinea pigs and humans lack gulono-gamma-lactone oxidase [17] and cannot synthesize vitamin C (VC); thus, these components must be obtained from food. In this study, VC was artificially administered to guinea pigs to study whether VC can activate vitamin D_3_ (VD_3_). DSS is widely used to study IBD, particularly in the DSS-induced UC model [18–20]. DSS destroys the intestinal mucosal barrier and causes intestinal substances to enter the intestinal tissue, inducing excessive activation of immune cells and triggering inflammatory reactions [21–23]. In this experiment, after drinking DSS solution, guinea pigs showed different degrees of weight loss, colonic inflammatory cell infiltration, and elevated colonic scores. Before DSS modeling, the guinea pigs were administered different doses of VC and VD_3_ by gavage every day, which protected the damaged colon of the guinea pigs to some extent.

The intestinal mucosal barrier is key to maintaining a balance between the intestinal lumen and mucosa, primarily through the mucous layer, intestinal epithelial cells, host innate immune system, and adaptive immune response [24]. To determine the effect of VC and VD_3_ on the colonic guinea pig colon in DSS, the ultrastructure of the colonic colon in guinea pigs was observed. The results revealed no significant change in the ultrastructure of the colonic epithelium in the control group. For the colonic epithelium of guinea pigs in the low, medium, and high dose VC groups, microvilli on the cell surface showed focal reduction, shortening, and sparseness to different degrees. The medium-dose VC group showed the most severe damage and the cell gap became larger, whereas the other two groups had a tightly connected structure. This suggests that VD_3_ can only protect the colon after DSS intervention when an appropriate combination of VC is administered.

The Notch signaling pathway is a highly conserved signaling pathway involved in the proliferation and differentiation of all cells. Notch signaling is associated with many human diseases [25] and is also closely related to IBD [26, 27], and thus is considered as a potential target for cancer treatment. Detection of genes related to the colon tissue in guinea pigs showed that low-dose VC strongly activated the Notch/Hes-1 signaling pathway. Combined with our electron microstructural ultrastructural results, a low dose of VC has some protective effect on the intestinal mucosa of guinea pigs with DSS-induced colitis. Studies have shown that when the intestinal epithelium is damaged, increased expression of Notch-1 can promote the proliferation of epithelial cells, which is conducive to the repair and reconstruction of the injured site; however, persistent overexpression of Notch-1 can lead to the reduction of intestinal epithelial secretory cell lines, which is not conducive for treating UC [28, 29]. Activation of the Notch signaling pathway alters the expression of tight junction proteins and affects the continuity of their distribution, thereby reducing cell barrier permeability. Studies have shown that vitamin D_3_ protects the gut barrier by regulating tight junction proteins [30]. We found that in the high-dose VC group, although the mRNA expression level of signal molecules in the Notch/Hes-1 pathway was significantly decreased, the expression level of the tight junction protein ZO-1 mRNA was increased; ZO-1 interacts with claudin-2 to correct the increase in permeability caused by disease [31], and high-dose VC significantly improved colonic tissue shortening caused by DSS in guinea pigs. Thus, when the same dose of VD_3_ was administered, a high dose of VC protected guinea pigs with DSS-induced colitis by reducing the increase in colonic mucosal permeability.

In addition, SW480 cells are derived from human colorectal adenocarcinoma cells and are one of the commonly used carriers for in vitro studies of intestinal inflammation. This study found that there was no significant difference in cell proliferation between the experimental groups. It can be seen that simple VD_3_ and different concentrations of VC combined with VD_3_ have no significant effect on the proliferation of SW480 cells induced by LPS. In the cell scratch experiment, it was found that VC combined with VD_3_ had better repair effects on cell scratches than VD_3_ alone. Based on a certain dose of VD_3_, 0.1 μmol/mL VC had the best repair effect on cell scratches, but with the dose of VC with the increase of VC, the ability of cell migration and repair has not been significantly improved, and when the concentration of VC reaches 10 μmol/mL, it has no obvious repair effect on cell scratches, indicating that VC combined with VD_3_ is more conducive to the repair of epithelial barrier damage, but it is not dose-dependent response relationship.

Our experimental study found that VD_3_ can antagonize the decrease in ZO-1 mRNA expression caused by LPS, but has no obvious inhibitory effect on the expression of claudin-2 mRNA. Compared with the D group, the expression of ZO-1 mRNA in the 5C + D group increased, while the expression of ZO-1 protein was not significantly different between the two groups. However, the expression of claudin-2 mRNA and protein in the 5C + D group was significantly lower than that in the D group, indicating that this concentration of VC combined with VD_3_ is more conducive to maintaining the intestinal epithelial cell barrier than VD_3_ alone, and mainly by inhibiting Claudin-2 generation plays a role.

## Conclusions

The effect of VC combined with VD_3_ on the damage repair of the cell barrier was better than that of VD_3_ alone, indicating that VC promotes the activation of VD_3_ to some extent. Appropriate application of VC combined with VD_3_ may alleviate damage to the colon mucosa and promote the repair of cell mucosal barrier damage. Although different concentrations of VC combined with VD_3_ had different effects on the levels of Notch signaling and tight junction proteins, the combination of VC and VD_3_ may reduce the expression of Notch-1 and inhibit the expression of claudin-2, thereby playing a protective role in the intestinal epithelial barrier.

## Data Availability

Data supporting the conclusions of this article are included in this article and in the supplementary materials.

## Abbreviations

DSS: Dextran sodium sulfate
VC: Vitamin C
VD_3_: Vitamin D_3_
LPS: Lipopolysaccharide
SW480 cell: Human colon cancer cells
